# Sulfonamide and tetracycline resistance genes in total- and culturable-bacterial assemblages in South African aquatic environments

**DOI:** 10.3389/fmicb.2015.00796

**Published:** 2015-08-04

**Authors:** Satoru Suzuki, Mitsuko Ogo, Tatsuya Koike, Hideshige Takada, Brent Newman

**Affiliations:** ^1^Center for Marine Environmental Studies, Ehime UniversityMatsuyama, Japan; ^2^Tokyo University of Agriculture and TechnologyFuchu, Japan; ^3^Coastal Systems Research Group, Natural Resources and the Environment, The Council for Scientific and Industrial ResearchDurban, South Africa

**Keywords:** antibiotic resistance, *sul*, *tet*(M), yet-to-be cultured, South Africa, sewage treatment plant

## Abstract

Antibiotic resistant bacteria are ubiquitous in the natural environment. The introduction of eﬄuent derived antibiotic resistance genes (ARGs) into aquatic environments is of concern in the spreading of genetic risk. This study showed the prevalence of sulfonamide and tetracycline resistance genes, *sul1, sul2, sul3*, and *tet*(M), in the total bacterial assemblage and colony forming bacterial assemblage in river and estuarine water and sewage treatment plants (STP) in South Africa. There was no correlation between antibiotic concentrations and ARGs, suggesting the targeted ARGs are spread in a wide area without connection to selection pressure. Among *sul* genes, *sul1* and *sul2* were major genes in the total (over 10^-2^ copies/16S) and colony forming bacteria assemblages (∼10^-1^ copies/16S). In urban waters, the *sul3* gene was mostly not detectable in total and culturable assemblages, suggesting *sul3* is not abundant. *tet*(M) was found in natural assemblages with 10^-3^ copies/16S level in STP, but was not detected in colony forming bacteria, suggesting the non-culturable (yet-to-be cultured) bacterial community in urban surface waters and STP eﬄuent possess the *tet*(M) gene. Sulfamethoxazole (SMX) resistant (SMX^r^) and oxytetracycline (OTC) resistant (OTC^r^) bacterial communities in urban waters possessed not only *sul1* and *sul2* but also *sul3* and *tet*(M) genes. These genes are widely distributed in SMX^r^ and OTC^r^ bacteria. In conclusion, urban river and estuarine water and STP eﬄuent in the Durban area were highly contaminated with ARGs, and the yet-to-be cultured bacterial community may act as a non-visible ARG reservoir in certain situations.

## Introduction

Antibiotic resistance genes (ARGs) are found not only in the clinical but also the natural environment, which can eventually produce antibiotic resistant bacteria (ARB). Antibiotics and ARB are released to the environment from hospitals, livestock facilities, and sewage treatment plants (STP) (Pruden et al., 2013). Although antibiotics are decomposed and diluted in the aquatic environment water, even at low concentrations they may act as signaling molecules in microbes (Fajardo and Martinez, 2008). Selection of ARG mutation by very low concentrations of antibiotics is reported (Gullberg et al., 2011). It is, therefore, critical to understand the fate of released antibiotics, ARB and ARGs in the environment, and whether residual ARGs in the environment pose a risk to humans. The aim of this study was to assess the status of antibiotics and ARGs in anthropogenically impacted surface waters in one area of South Africa.

The status of antibiotic use and STP operation differs between countries. Consequently, the status of antibiotic contamination and presence of ARBs and ARGs in aquatic ecosystems must be established on a case by case basis. In previous monitoring we showed the status of antibiotic contamination (Shimizu et al., 2013) and ARGs (Suzuki et al., 2013) in numerous Asian countries. In many tropical Asian countries an integrated system of agriculture is followed, which includes animal husbandry, aquaculture, and crop farming (Suzuki and Hoa, 2012). In this system the major antibiotic used for animals is sulfonamides. Tetracyclines are also used in aquaculture. STPs receive wastewater and excreta from humans and livestock facilities, which intimates the mixing of waters containing various antibiotics, ARB and ARGs. The main purpose of conventional STPs is to prevent the spread of infectious diseases and reduce solid and nutrient loads from excreta entering surface waters, not to decompose pharmaceuticals and genes. Although advanced disinfection technologies can greatly reduce the danger of waterborne diseases (United States Environmental Protection and Agency, 2004), antibiotics and ARGs are not completely decomposed in the STP process and are released into the environment (Rizzo et al., 2013; Berkner et al., 2014).

The populations and economies of African countries are developing. Although South Africa has a relatively well developed economy by African standards, many STPs are not functioning efficiently and are overloaded and has been identified as a serious cause for concern (Snyman et al., 2006; Water Research and Commission, 2006). Furthermore, most South African cities are characterized by large informal settlements where sanitation facilities are poor and in some cases essentially non-existent, with pit latrines and mobile toilets usually the only form of sanitation. This might result in the introduction of antibiotics, ARB and ARGs into the aquatic environment. Omulo et al. (2015) reviewed many articles on ARB research from Eastern Africa, which mainly studied on human and animal bacteria. Environmental ARB needs to be studied further.

It is well known that the majority of bacteria in aquatic environments are non-culturable or yet-to-be cultured bacteria (Bloomfield et al., 1988; Amann et al., 1995; Takami et al., 2009). In recent monitoring in the Philippines we showed that the total bacterial community in seawater possessed minor sulfonamide resistance gene *sul3*, which was not detected in colony forming bacteria (Suzuki et al., 2013). This suggests that the abundant non-culturable or yet-to-be cultured bacteria in aquatic environments are a reservoir of ARGs, but these are not detectable by culture methods. The *sul3* gene was detected in human and non-human isolates of *Salmonella* in Portuguese waters, although *sul3* was a minor contributor compared to *sul1* and *sul2* genes (Antunes et al., 2005). In Denmark, *Escherichia coli* isolated from pork and pigs possessed *sul3*, but this gene was not found in human isolates (Hammerum et al., 2006). In Germany, *sul3* was not found in *E. coli* of human isolates, but was found in cattle, pig, and poultry isolates (Guerra et al., 2003). These studies suggest that *sul3* is spreading widely amongst animals but not amongst humans, possibly due to the use of sulfonamide for animal husbandry but not in humans in developed countries, and that *sul3* is transferred by a different gene cassette to *sul1* and *sul2* (Antunes et al., 2005). Although recent advances in metagenomics can detect total resistome, quantitative estimation of ARGs in the microbial community is not yet possible. An understanding of the reservoir of culturable- and non-culturable bacteria in the environment might thus be useful in assessing whether environmental ARGs are posing a risk.

The aim of this study was to assess the abundance of *sul1, sul2, sul3*, and *tet*(M) genes in total- and colony forming-bacterial assemblages in surface waters and STP eﬄuent in the eThekwini area of South Africa. As far as we are aware, no information in this context is available for this area, or indeed for other areas in South Africa. Sulfonamides and tetracyclines have a long use as human and animal therapeutic agents and animal growth promoters. Sulfonamide resistance occurs mainly by mutation of the dihydropteroate synthase (DHPS) gene, although other mechanisms are known (Radstrom and Swedberg, 1988; Huang et al., 2004). As to tetracycline resistance, 45 *tet* genes are known at this time (Roberts et al., 2012). Among the *tet* genes, *tet*(M), a ribosomal protection protein gene is suspected of having the broadest host range (Roberts et al., 2012) and its origin is reported to be ancient (Kobayashi et al., 2007). Additionally, *tet*(M) shows high genetic diversity (Rizzotti et al., 2009) and wide distribution in the natural environment (D’Costa et al., 2011). Therefore, we focused on the *sul* genes and *tet*(M) as monitoring targets. We hypothesized that eﬄuent from inefficient STPs or wastewater derived from poor sanitary conditions should contain high concentrations of ARGs from human bacteria. The comparison of ARGs using culture-dependent and independent methods should, therefore, provide an understanding on whether bacterial communities of natural or human origin are the major reservoir of ARGs in aquatic ecosystems.

## Materials and Methods

### Sampling of Water

**FIGURE 1:**
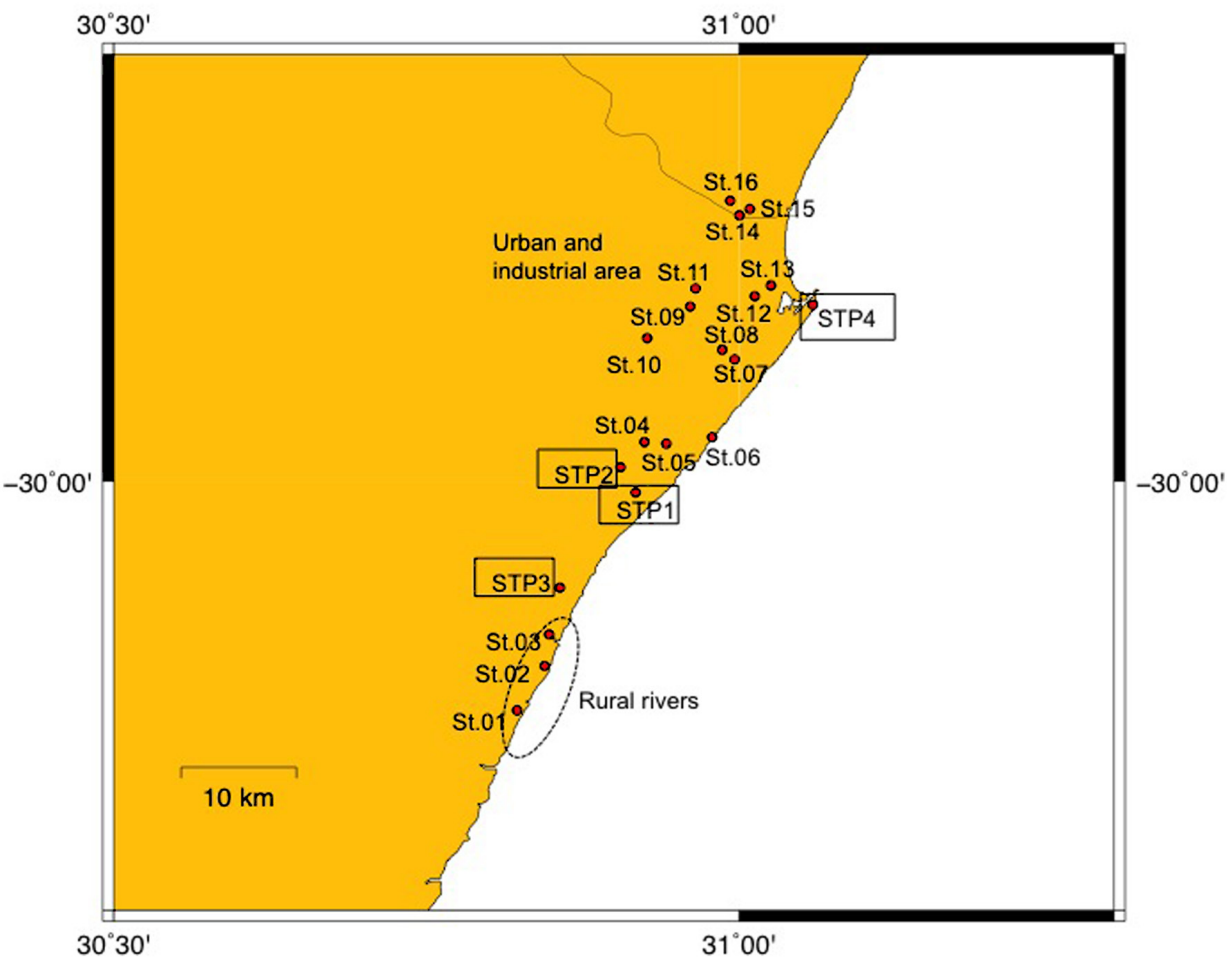
**Map of sampling sites.** Sites 01–03 are rural river sites, Sites 04–16 are urban river sites, and STP1-4 are sewage treatment plants (STPs).

Samples were collected with an ethanol rinsed stainless steel bucket between September 3 and 5 in 2012, in the eThekwini Metropolitan Municipality area in the province of KwaZulu-Natal, on the subtropical northeast cost of South Africa (**Figure 1**). Characteristics of the sampling sites are summarized in Supplementary Table S1. The municipality has a population size of about 3,400,000 (Statistics South Africa, http://www.statssa.gov.za/?page_id=1021&id=ethekwini-municipality). The city of Durban and a number of smaller towns fall in the municipal area. Rainfall in the eThekwini area is seasonal, falling predominantly in summer. Although it was not raining at the times that samples were collected, about 31 mm of rain was recorded at rain monitoring gage in Durban during the sampling period. Because samples were collected at a single point in time we recognize this study does not provide an understanding on the temporal variability of ARBs and ARGs in surface waters and STP eﬄuents in the study area.

Surface water samples were taken at three sites (Sites 01–03) in the estuarine parts of rivers situated in rural locations. Thirteen sites (Sites 04–16) were sampled in the riverine and estuarine parts of rivers with urbanized and industrialized catchments in the greater Durban area, and eﬄuent was collected from four STPs (STP1–4). The water and eﬄuent was filtered through 50 μm mesh plankton net to remove large debris, and stored on ice for a few hours until analysis. Further detail on the condition of surface waters at the river and estuarine sites is provided in Segura et al. (2015). Water samples indicate present status of contamination (Takasu et al., 2011).

### Antibiotic Concentration

Sulfonamides and tetracyclines were analyzed using a liquid chromatograph (Accela, Thermo Scientific) equipped with a tandem mass spectrometer (LC-MS/MS; Quantum Access, Thermo Scientific) after extraction using a solid-phase cartridge (Oasis HLB resin, Waters). The analytical process was the same as that provided in Segura et al. (2015).

### Bacterial Count

Total bacterial cell number was counted by DAPI staining according to Sato-Takabe et al. (2015). Total viable count and sulfamethoxazole resistant (SMX^r^) and oxytetracycline resistant (OTC^r^) bacterial numbers were enumerated on nutrient agar plates (LB plus 1.5% agar) incubated at 30°C for 24 h. To estimate SMX^r^ and OTC^r^ bacteria, 60 μg/mL of each drug was supplemented to the medium (Hoa et al., 2011). All plate counts were performed in duplicate.

### Quantitative Analysis of Antibiotic Resistance Genes (ARGs)

The sulfonamide resistance genes, *sul1, sul2*, and *sul3*, and tetracycline resistance gene, *tet*(M), were quantified by quantitative PCR (qPCR) from total assemblage using total DNA trapped on 0.2 μm pore filter. For the culturable bacterial assemblage, all colonies on agar plates were mixed and used for qPCR. DNA extraction from the filter and mixtures of colonies was previously reported (Suzuki et al., 2013). DNA from filters and colonies were obtained from triplicate biological samples. qPCR was performed using a CFX 96 Real-Time system (BioRad, Laboratories, Hercules, CA, USA) to detect an increase of double-stranded DNA with an increase in fluorescence according to Suzuki et al. (2013). PCR amplifications were performed in a 20 μl reaction volume containing 1 X Sso Fast EvaGreen Supermix (Bio-Rad), 500 nM of each primer and 1 μl of sample DNA. qPCR was performed using previously designed primers; bacterial 16S rRNA genes (Suzuki et al., 2000), *sul1* (Heuer and Smalla, 2007), *sul2* (Heuer et al., 2008), *sul3* (Pei et al., 2006), and *tet*(M) (Tamminen et al., 2011). Serial 1:10 dilutions of plasmids constructed from the pGEM-T Easy vector (Promega, Madison, WI, USA) and 16S rRNA gene from *E. coli* K12, *sul1* from plasmid R388, *sul2* from plasmid RSF1010, *sul3* from plasmid pUVP4401 (Heuer and Smalla, 2007), and *tet*(M) from pFD310 fragments (Smith et al., 1992) were used as standards for quantification. The qPCR program consisted of an initial denaturation of 30 s at 95°C and 40 cycles of 5 s at 95°C and 10 s at 50°C for 16S rRNA gene and 10 s at 51°C for *sul1* and *sul2* and 20 s at 60°C for *sul3*, and 20 s at 57°C for *tet*(M), respectively. Melting curves for the amplicons were measured by raising the temperature slowly from 60°C and 65°C to 95°C for 16S rRNA gene, *sul1, sul2, sul3, tet*(M), and *sul3*, respectively, while monitoring fluorescence. Each sample was measured in triplicate. The copy numbers of *sul1, sul2, sul3*, and *tet*(M) were normalized by dividing by the 16S rRNA gene copy number at the respective time points to take into account any temporal variation in bacterial cell numbers. Unit of the copy number is described as copies/16S in the text. The results were analyzed using a Big Dye terminator kit on a 3130 ABI Prism sequencer (Applied Biosystems, Foster City, CA, USA). PCR products were sequenced to confirm they were not non-specific products.

## Results and Discussion

### Drug Contamination

The distribution of antibiotic concentrations in surface waters and STP eﬄuent showed that SMX was a major contaminant along with trimethoprim, which is a combination drug. The SMX concentrations were: rural surface waters - 48.2 ± 71.2 ng/L (*n* = 3), urban surface waters - 2561 ± 51.3 ng/L (*n* = 13), STP eﬄuent - 3612 ± 1733.4 ng/L (*n* = 4). High SMX concentrations in urban surface waters and STP eﬄuent indicate its frequent use in human chemotherapy. It is also reported that SMX is frequently used in African countries to control bacteria and protozoan infections in HIV patients (Zachariah et al., 2007). Recently report in Ghana, Mozambique, Kenya, and South Africa showed that the SMX is the highest concentration among selected 18 antibiotics in all countries (Segura et al., 2015). Data from STP in the present study showed high concentration compared to these. Tetracyclines were mostly not detectable in surface waters and STP eﬄuent (maximum 18 ng/L, and mostly below detection limit). At one STP (STP4), however, 291 ng/L of OTC was detected, indicating real time use of the drug. The results suggest that SMX is used frequently in the Durban area. The concentration over 1000 ng/L was similar to a pig farm in Vietnam (Hoa et al., 2011; Shimizu et al., 2013), and double that of STP eﬄuent in Michigan, U. S. (Gao et al., 2012b). Erythromycin (1194 ng/L) was also present in STP4 eﬄuent, but was not particularly prevalent in surface water samples, suggesting the antibiotics originated from human medicines. The high contamination of surface waters and STP eﬄuents by antibiotics suggests that ARGs in hospitals are also likely entering the environment (Pruden, 2014).

### Bacterial Numbers

The counts of bacteria in different surface water and STP eﬄuent samples are shown in **Table 1**, as enumerated by DAPI count (total number), plate count (colony forming number), and SMX^r^ and OTC^r^ bacterial counts. Total cell number was almost the same in the rural and urban surface waters, with 10^6^ cells/ml, but an order of magnitude higher in STP eﬄuents. The colony forming number was two orders of magnitude lower than the total cell number. The contribution of culturable bacteria to the total cell number was 1.0–1.5% in surface waters and 6.8% in STP eﬄuents, a statistically significant difference (*p* < 0.05, *t*-test). The culturable bacterial contribution to the total cell number in freshwater is reported to be approximately 0.25% (Amann et al., 1995), indicating that the number of culturable bacteria was higher in surface waters and STP eﬄuents in Durban. Dominance rate of viable number was higher in urban surface waters and STP eﬄuent compared to rural surface waters, suggesting contamination of culturable bacteria is derived from human sources. Iweriebor et al. (2015) reported in South Africa that resistance rate of *Enterococcus* from hospital and STP eﬄuents was 67–100%. The contribution of ARB in STP eﬄuent was higher than in surface waters in our study (SMX^r^, *p* < 0.05 and OTC^r^, *p* < 0.01). Culturable bacteria in STP eﬄuent should include enteric bacteria, which form colonies on agar plates with a contribution of 15% (Langendijk et al., 1995) compared to 0.1% in seawater (Amann et al., 1995; Fuhrman and Hagström, 2008). Abundances of SMX^r^ and OTC^r^ bacteria were not positively correlated to antibiotic concentrations. It is reported that drug concentrations and occurrence of ARB are not correlated to fluoroquinolones in environment (Takasu et al., 2011). Although the reason why sulfonamide- and tetracycline-resistance are frequently found in non-contaminated environments is not known, the heavy use of sulfonamides and tetracyclines in the 20th century could be one of the reasons for the selection of SMX^r^ - and OTC^r^ -genes in bacterial communities. Sediment stores *sul* and *tet* genes for a long time in non-contaminated areas (Tamminen et al., 2011; Muziasari et al., 2014), whereas water samples indicate present status. The abundance of ARB in surface waters suggests their continuous input into the environment. The ARGs for these drugs should be distributed in various environmental bacteria around the world. There are factors other than antibiotics, such as metals (Knapp et al., 2011), that may select for ARB and ARGs in natural bacterial assemblages.

**Table 1:** Bacterial number in three categorized sites.

Site	Total cell count (cells/ml)	Colony count (CFU/ml) (% of total)	Sulfamethoxazole resistant (SMX^r^; CFU/ml) (% of colony count)	Oxytetracycline resistant (OTC^r^; CFU/ml) (% of colony count)
Rural river (*n* = 3)	(1.1 ± 0.47) × 10^6^	(1.1 ± 1.4) × 10^4^ (1.0%)	(1.8 ± 3.0) × 10^3^(16.8%)	(5.0 ± 7.0) × 10^2^(4.7%)
Urban and industrial river (*n* = 13)	(2.7 ± 2.7) × 10^6^	(4.1 ± 4.8) × 10^4^(1.5)	(8.1 ± 7.7) × 10^3^ (20.0)	(4.8 ± 6.9) × 10^3^(11.9)
Sewage treatment plant (STP) (*n* = 4)	(1.0 ± 1.0) × 10^7^	(7.0 ± 11) × 10^5^(6.8)	(2.1 ± 3.0) × 10^5^ (30.4)	(4.8 ± 5.1) × 10^4^(6.9)

### The *sul* and *tet*(M) Genes in Total- and Culturable-Assemblages

**FIGURE 2:**
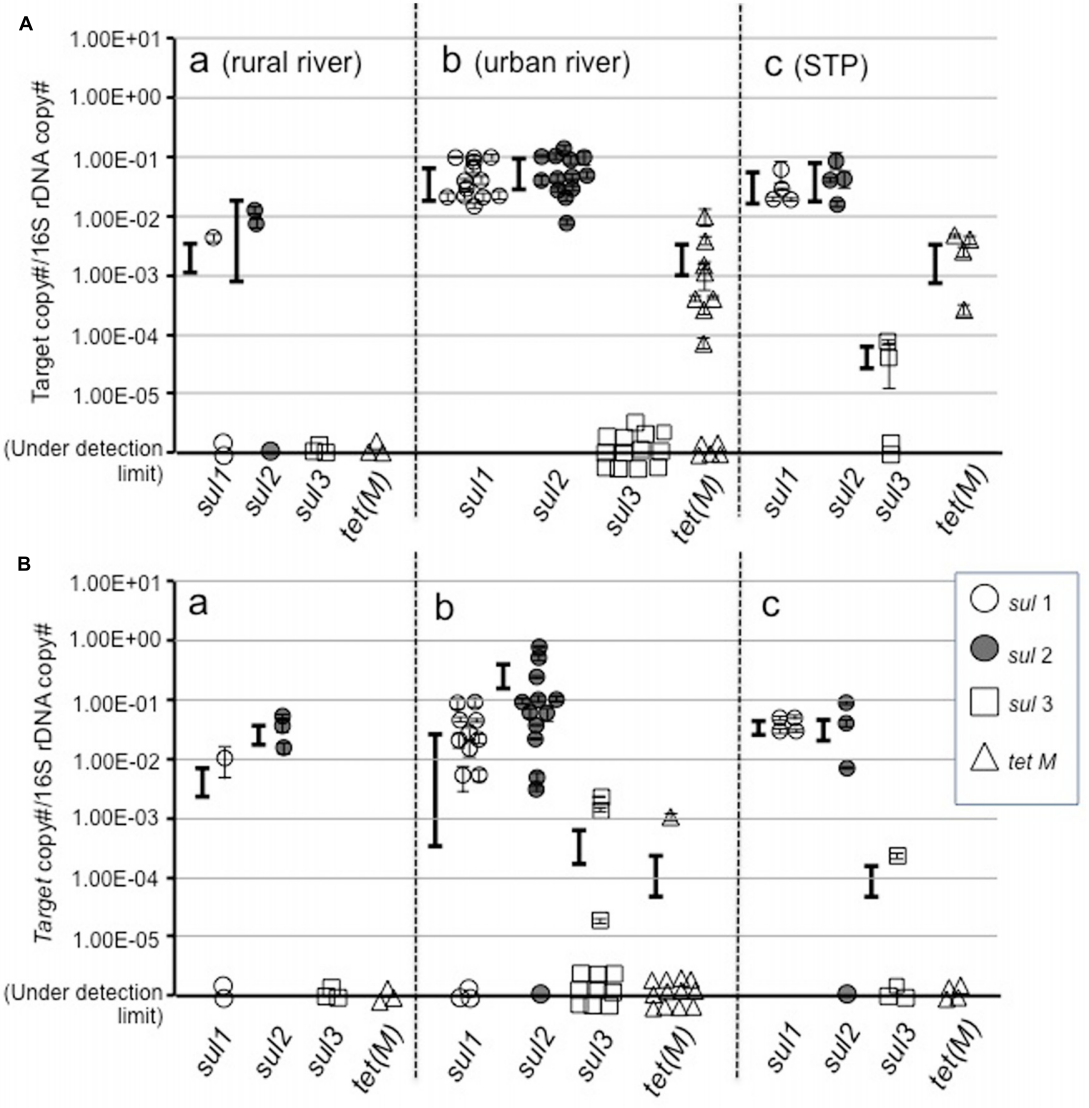
**Abundance of *sul1, sul2, sul3*, and *tet*(M) genes in total **(A)** and culturable bacterial assemblages (B)**.

Among *sul* genes, *sul1* and *sul2* were detected at a similar copy number in total assemblages in all categories of water (**Figure 2A**), and also in colony forming bacteria (**Figure 2B**). This indicates that *sul1* and *sul2* are ubiquitous in bacterial communities, including yet-to-be cultured and culturable bacteria in aquatic environments in the Durban area. At most urban river and estuarine and STP sites, *sul1* and *sul2* were present at copy numbers of 10^-2^–10^-1^/16S. These values are higher than at rural sites in the Durban area, and in the Philippines (Suzuki et al., 2013) and in Finnish sediment (Muziasari et al., 2014), but are comparable to values reported for suspended solids in lagoon waters (McKinney et al., 2010).

Profiles for *sul3* and *tet*(M) were different from *sul1* and *sul2* between total assemblage and culturable bacteria. The *sul3* gene was not detected or was at a very low abundance in the total assemblage at most sites, although two sites showed 10^-5^–10^-4^/16S. In the case of the Philippines, *sul3* was not detected in natural assemblages or colony forming bacteria in freshwater lakes and rivers, whereas a high copy number was detected in seawater assemblages (Suzuki et al., 2013). The fact that *sul3* was not at a high copy number in culturable bacteria (**Figure 2B**) suggests this gene is not abundant in the Durban area. Gao et al. (2012a) reported similar results in freshwater.

In the case of *tet*(M), the total assemblage in urban surface waters and STP eﬄuents possessed approximately 10^-3^/16S, whereas culturable bacteria did not. This suggests the yet-to-be cultured community possesses *tet*(M). Since the yet-to-be cultured bacteria comprise the major component of the bacterial community, the gene pool of *tet*(M) in environment should be large. A risk assessment for ARGs amongst this silent majority is required.

**FIGURE 3:**
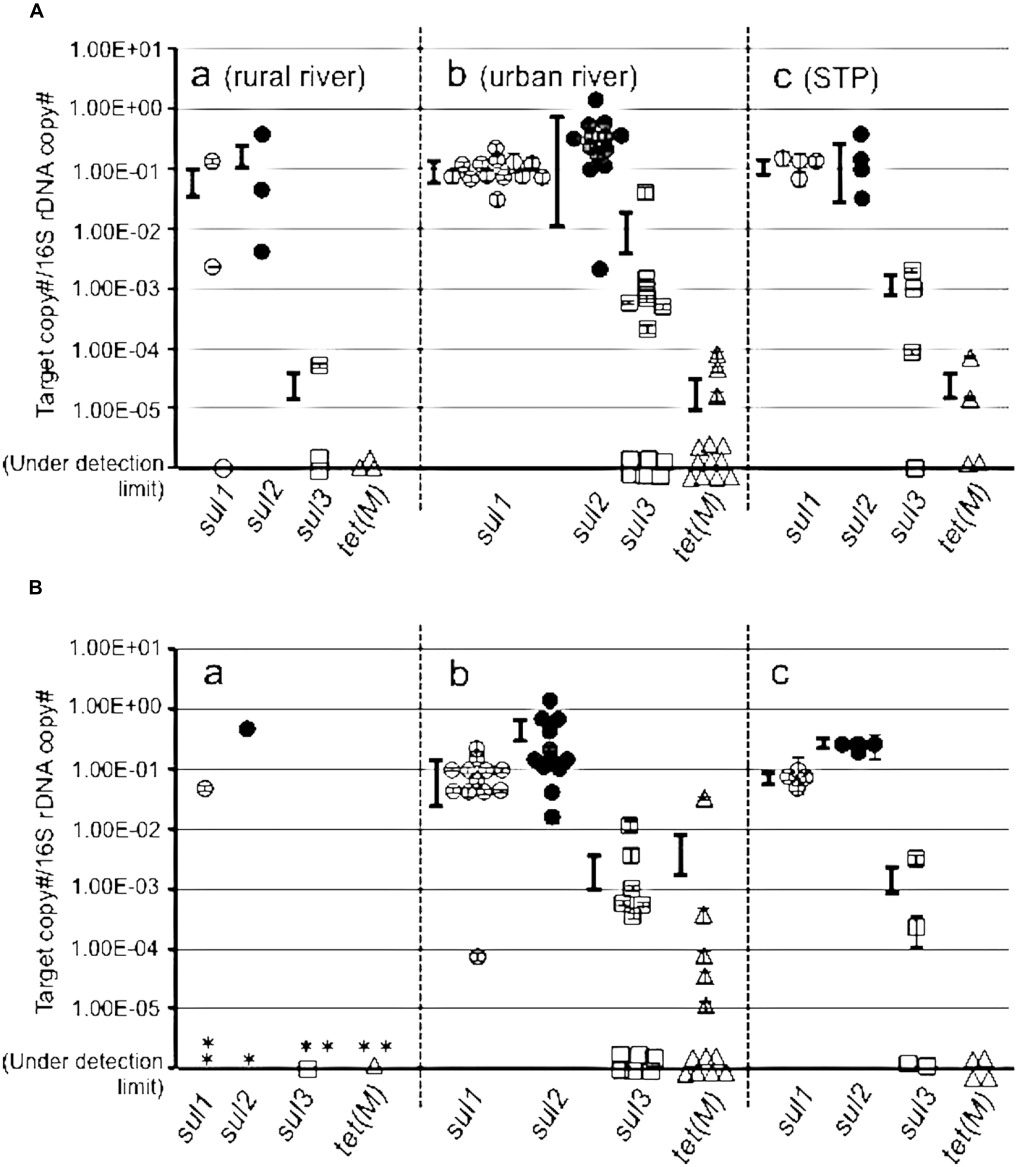
**Abundance of *sul1, sul2, sul3*, and *tet*(M) genes in sulfamethoxazole resistant (SMX^r^) colonies mix **(A)** and in oxytetracycline resistant (OTC^r^) colonies mix (B). (B)** Asterisk shows sites where resistant isolate was not obtained. Symbols are the same to **Figure 2**.

The copy numbers of the targeted ARGs were measured in a pooled colony from SMX^r^ and OTC^r^ bacteria (**Figure 3**). The *sul1* and *sul2* were higher than 10^-1^/16S at urban and STP sites, with *sul3* around 10^-3^∼10^-2^/16S (**Figure 3A**). The rural sites also showed high copies of *sul1* and *sul2*, but *sul3* was detected at only one site at a low concentration. This indicates that colony forming SMX^r^ bacteria possess *sul* genes, which were selected on SMX-containing agar plate. The *sul* genes in the OTC^r^ assemblage also showed a high copy number of *sul* genes (**Figure 3B**). It is reported that *sul* and *tet* genes are sometimes coded on the same plasmid of aquatic bacteria (Kim et al., 2008; Nonaka et al., 2012), and SMX^r^ and OTC^r^ phenotypes are frequently linked (Hu et al., 2008). The present study supports the findings in terms of gene copy numbers in assemblages by cross checking with SMX^r^ and OTC^r^ bacteria. On the other hand, *tet*(M) copy number was less than 10^-2^ in SMX^r^ and OTC^r^ bacteria at most sites, suggesting two possibilities. One is that the selected bacteria by SMX and OTC possess other *tet* genes than *tet*(M), and is the other that *tet*(M) is abundant in total assemblages but not in culturable resistant bacteria.

## Conclusion

Quantitative PCR and culture methods revealed that *sul* genes are conveyed by bacterial communities in urban surface waters and STP eﬄuent in the Durban area of South Africa. Additionally, *sul3* was detected in the culturable bacteria assemblage. The yet-to-be cultured bacterial community may act as a non-visible reservoir of ARGs in certain situations.

## Conflict of Interest Statement

The authors declare that the research was conducted in the absence of any commercial or financial relationships that could be construed as a potential conflict of interest.
