# Supplementary material for: Sulfonamide and tetracycline resistance genes in total- and culturable-bacterial assemblages in South African aquatic environments
Source: Front Microbiol. 2015 Aug 4;6:796. doi: 10.3389/fmicb.2015.00796 (PMC4523819; doi:10.3389/fmicb.2015.00796)
Supplement: Supplementary file 1 [file Table_1.DOCX]

Table S1. Characters of the sampling sites

| Site | River name (STP name) | Water temp (˚C) | pH | EC (µS/cm) | Salinity | Depth (m) | Description |
| --- | --- | --- | --- | --- | --- | --- | --- |
| St.01 | uMgababa | 20.8 | 7.79 | 25600 | 20 | 1.5 | Catchment largely in Tribal Trust land and dominated by small scale/subsistence type farming. Most dwellings with pit or soak away latrines. |
| St.02 | Msimbazi | 21.9 | 7.72 | 21000 | 12 | 2.5 | ditto |
| St.03 | Lovu | 22.2 | 6.83 | 18660 | 10 | 0.5 | Large part of catchment in Tribal Trust land and dominated by small scale/subsistence type farming but also some commercial scale farming. Most dwellings with pit or soak away latrines, but near mouth dwellings and so on connected to sewer system. |
| St.04, | Isipingo | 19.7 | 7.06 | 935 | nd | 0.3 | Highly industrialised catchment |
| St.05 | Isipingo | 21.6 | 7.13 | 2340 | 0 | 0.8 | ditto |
| St.06 | uMlaas | 24.1 | 9.77 | 624 | 0 | 0.1 | ditto |
| St.07 | Amanzimnyama | 23.9 | 9.54 | 563 | 0 | 0.5 | ditto |
| St.08 | uMhlatuzana | 24.3 | 8.59 | 482 | 0 | 0.3 | Highly urbanised catchment, with some industry |
| St.09 | uMbilo | 22.7 | 8.04 | 581 | 0 | 0.2 | ditto |
| St.10 | uMbilo | 19.5 | 7.18 | 453 | 0 | 0.2 | ditto |
| St.11 | uMkumbaan | 17.5 | 7.76 | 712 | 0 | 0.2 | Highly urbanised catchment, with large informal settlements with poor sanitation facilities (pit latrines and portable toilets) |
| St.12 | Unknown | 18.5 | 7.07 | 21300 | 12 | nd | Highly industrialised catchment, close to sea |
| St.13 | Unknown | 18.4 | 7.16 | 32300 | 20 | nd | ditto |
| St.14 | Palmiet | 18.5 | 7.94 | 603 | nd | 0.1 | Highly urbanised and industrialised catchment |
| St.15 | Unknown | 19.9 | 6.79 | 17560 | 10 | nd | ditto |
| St.16 | Ottawa Stream | 19.1 | 7.29 | 663 | 0 | nd | ditto |
| STP1 | Amanzimtoti STP | 19.3 | 7.10 | 1010 | 0 | nd | Domestic and some industrial effluent (Discharged into river) |
| STP2 | Isipingo STP | 18.1 | 6.70 | 528 | 0 | nd | ditto |
| STP3 | Kingsburgh STP | 18.0 | 7.26 | 647 | 0 | nd | Domestic effluent (discharged into river) |
| STP4 | Central STP | 20.5 | 7.06 | 1047 | 0 | nd | Domestic and some industrial effluent (Discharged to sea) |

nd, not determined
